# Undergraduate medical students’ perceptions and intentions regarding patient safety during clinical clerkship

**DOI:** 10.1186/s12909-018-1180-8

**Published:** 2018-04-04

**Authors:** Hoo-Yeon Lee, Myung-Il Hahm, Sang Gyu Lee

**Affiliations:** 1 Department of Social Medicine, College of Medicine, Dankook University College of Medicine, Cheonan, Republic of Korea; 2 Department of Health Administration and Management, College of Medical Science, Soonchunhyang University, Asan, Republic of Korea; 3 Department of Hospital management, Graduate School of Public Health, Yonsei University, Seoul, Republic of Korea

**Keywords:** Patient safety, Safety culture, Medical students

## Abstract

**Background:**

The purpose of this study was to examine undergraduate medical students’ perceptions and intentions regarding patient safety during clinical clerkships.

**Methods:**

Cross-sectional study administered in face-to-face interviews using modified the Medical Student Safety Attitudes and Professionalism Survey (MSSAPS) from three colleges of medicine in Korea. We assessed medical students’ perceptions of the cultures (‘safety’, ‘teamwork’, and ‘error disclosure’), ‘behavioural intentions’ concerning patient safety issues and ‘overall patient safety’. Confirmatory factor analysis and Spearman’s correlation analyses was performed. In total, 194(91.9%) of the 211 third-year undergraduate students participated**.**

**Results:**

78% of medical students reported that the quality of care received by patients was impacted by teamwork during clinical rotations. Regarding error disclosure, positive scores ranged from 10% to 74%. Except for one question asking whether the disclosure of medical errors was an important component of patient safety (74%), the percentages of positive scores for all the other questions were below 20%. 41.2% of medical students have intention to disclose it when they saw a medical error committed by another team member.

**Conclusions:**

Many students had difficulty speaking up about medical errors. Error disclosure guidelines and educational efforts aimed at developing sophisticated communication skills are needed. This study may serve as a reference for other institutions planning patient safety education in their curricula. Assessing student perceptions of safety culture can provide clerkship directors and clinical service chiefs with information that enhances the educational environment and promotes patient safety.

## Background

In an increasingly complex and fragmented healthcare system, the quality and safety of care have become major foci of leaders in the field, the public, regulatory entities, and accrediting bodies [1]. Indeed, quality and safety have become significant and influential considerations not only in the practice of healthcare but also in the education of practitioners. Developing a culture of safety is a core element of many efforts to improve patient safety and care quality [2]. Several studies have shown that the safety culture is associated with clinician behaviours in terms of increased error reporting, reductions in adverse events, and reduced mortality. Speaking up about safety issues without fear of blame or retribution, having feedback mechanisms in place to explain any improvements made after safety issues have been addressed, and promoting transparency are key to building a strong safety culture [3]. These major elements of patient safety science are often not taught explicitly but rather are covered as part of general training in clinical methods.

Educators must begin to recognise the potential effects of the informal and hidden curriculum encountered by trainees as they leave the classroom and enter the clinical environment that serve to implicitly undermine what is explicitly taught and must create mechanisms to counteract their influences [4]. The hidden curriculum consists of what is implicitly taught by example day to day, not the explicit teaching of lectures, grand rounds, and seminars [5, 6]. The hidden curriculum refers to medical education as more than simple transmission of knowledge and skills; it is also a socialization process. Research on this hidden curriculum has shown that there are frequently stark differences between what medical educators say and what they do when acting as role models for students and, in turn, between what students are explicitly taught and what they actually learn [7].

Recently, several policies related to patient safety issues have received attention in Korea. The Patient Safety Act was implemented in July 2016 in Korea, and patient safety indicators and standards were also introduced [8]. This change in the healthcare policy environment included a requirement to incorporate patient safety principles into medical education, including at the undergraduate level. As efforts to include patient safety in health professional education have increased, it has become increasingly important to understand the perspectives of trainees and new healthcare professionals on their own patient safety knowledge and attitudes [9, 10]. Several studies on patient safety education have focused primarily on healthcare providers, especially on more senior hospital doctors and nurses rather than undergraduate medical students [11–17]. Indeed, these issues have hardly been investigated in the context of undergraduate medical education in Korea [18].

The purpose of this study was to determine medical students’ perceptions and intentions regarding patient safety during clinical clerkships. The objectives were as follows: 1) to examine the perceptions with regard to safety, teamwork, and error disclosure of undergraduate medical students performing clinical rotations and to assess their intentions about patient safety, and 2) to analyse correlations between each domain and students’ intentions regarding patient safety.

## Methods

### Participants and survey administration

In general, the medical curriculum in South Korea is similar to those of U.S. and Canadian medical schools [19]. However, most Korean medical schools are undergraduate schools where students receive a total of six years of medical training comprising two years of premedical curriculum and four years of medical curriculum. The four years of medical curriculum comprise two years of preclinical work followed by two years of clinical work.

This cross-sectional study collected data via surveys administered in face-to-face interviews with 3rd-year undergraduate students enrolled at three colleges of medicine in Korea. All three schools were private and located in urban areas. The study was conducted in the fall of 2015, when the students were participating in clerkships in clinical settings. A clerkship is an opportunity for a small group of students to participate directly in the management of clinical problems presented by patients in hospitals. Clinical lectures are also given during this period.

Of the 211 third-year students invited to participate in the survey, 194 (91.9%) were finally included in the analysis after excluding students with missing variables; 39/39 (100%) at school A, 41/46 (89.1%) at school B, and 114/126 (90.5%) at school C. The study was approved by the Institutional Review Board of Yonsei University Graduate School of Public Health (2–1,040,939-AB-N-01-2015-305).

### Questionnaire

The questionnaire addressed five domains with 25 questions. We modified and translated the Medical Student Safety Attitudes and Professionalism Survey (MSSAPS) to measure Korean students’ perceptions of the cultures surrounding safety, teamwork, and error disclosure; to assess their behavioural intentions concerning patient safety issues; and to derive a grade for overall patient safety [7]. All items were rated on a five-point Likert scale. Students were asked to respond to the statement using a five-point Likert scale ranging from 1 (Strongly Disagree) to 5 (Strongly Agree).

### Statistical analysis

Because the survey items were adapted from other sources, we performed confirmatory factor analysis with the SAS procedure ‘PROC CALIS’ to confirm that the hypothesised model provided a good fit with the data [7]. The value of the Root Mean Square Error of Approximation(RMSEA) was ≤0.08. The Comparative fit index (CFI), Normed Fit Index (NFI), and Nonnormed Fit Index (NNFI) were ≥ 0.95. Thus, the fit statistics were acceptable [20–22]. The CFI identified the safety culture (9 items), teamwork culture (5 items), error disclosure culture (5 items), and behavioural intentions regarding patient safety (5 items).

Ratings on the five-point Likert scale were collapsed into “agree and strongly agree” as a positive response after considering reverse-coded negatively worded items, such as “If I saw a medical error committed by one of my team members, I would keep it to myself.” In terms of the overall patient safety grade, which was based on a 10-point Likert scale, scores from 7 to 10 were considered positive responses, and scores from 0 to 6 were grouped as negative responses [7].

Associations were assessed using Spearman’s coefficient and 95% confidence intervals (CIs) between safety culture, teamwork culture, error disclosure culture, and behavioural intent regarding safety and overall perception of patient safety. Analyses were performed using the SAS software (ver. 9.2; SAS Institute, Cary, NC, USA).

## Results

### Participants

In total, 194 medical students participated in the survey; 70.6% were males, and 29.4% were females (Table 1); their mean age was 24.3 years.

**Table 1:** Characteristics of survey participants

Medical school	Overall	Males	Age
A	39(20.1%)	28(71.8%)	23.4(±0.8)
B	41(21.1%)	26(63.4%)	24.4(±2.0)
C	114(58.8%)	83(72.8%)	24.6(±2.0)
Total	194(100.0%)	137(70.6%)	24.3(±1.9)

### Medical students’ perceptions and intentions

There were substantial variations in the percentages of positive scores across items in each domain (Table 2). The positive scores for the safety culture domain ranged from 33% to 89%. Over three-quarters of respondents agreed they followed standard operating procedures, guidelines, and protocols for the floor and operating room. The percentages of positive scores were low for appropriate feedback about performance and the clinical culture regarding learning from the errors of others.

**Table 2:** Medical students’ perceptions and intentions regarding patient safety

			Score (1–5) 1 (Strongly disagree) to 5 (strongly agree)				% positive responses	
Domain		Item	Mean	SD	Median	IQR	*n*	%
Safety culture	1	I received appropriate feedback about my performance	3.0	1.0	3	2	64	33.0
	2	We followed standard operating procedures, guidelines, and protocols for the floor (e.g. checklists to prevent central blood stream infections, hand washing)	4.0	0.9	4	2	143	73.7
	3	I observed excellent patient safety practices	3.8	0.9	4	1	132	68.0
	4	Medical errors were handled appropriately	3.7	0.9	4	1	116	59.8
	5	I was encouraged by colleagues to report any patient safety concerns I may have had	3.7	1.1	4	2	110	56.7
	6	The clinical culture made it easy to learn from the errors of others	3.1	1.1	3	2	67	34.5
	7	I would have felt safe being treated here as a patient	3.4	1.0	3	1	96	49.5
	8	I knew the proper channels to direct questions regarding patient safety	3.2	1.2	3	2	87	44.8
	9	We followed standard operating procedures, guidelines, and protocols for the OR (e.g. preoperative briefings, adherence to sterile technique for surgery and procedures)	4.5	0.7	5	1	173	89.2
Teamwork culture	1	The quality of care received by patients was impacted by teamwork	4.0	0.9	4	1	152	78.4
	2	I had good collaboration with nurses	3.7	0.9	4	1	124	63.9
	3	I had good collaboration with team members (students, residents, attending, nurses, and other caregivers)	3.8	0.8	4	1	127	65.5
	4	Disagreements were resolved appropriately (i.e. by emphasising not who is right but what is best for the patient)	3.7	0.9	4	1	125	64.4
	5	It was easy for personnel to ask questions when there was something that they did not understand	3.2	1.1	3	2	78	40.2
Error disclosure culture	1	When errors were made, they were disclosed to patients/families	2.8	1.0	3	1	48	24.7
	2	The culture during my rotations made it easy to disclose medical errors	2.5	1.0	2	1	26	13.4
	3	I was encouraged by my colleagues to disclose errors to patients/families	2.4	1.0	2	1	20	10.3
	4	I received education or training on how to disclose medical errors to patients	2.4	1.1	2	2	35	18.0
	5	Medical error disclosure to patients and families was an important component of patient safety	4.0	0.9	4	2	143	73.7
Safety behavioural intent	1	I expect to participate in quality improvement initiatives	3.8	0.8	4	1	118	60.8
	2	I intend to encourage my colleagues to tell patients and their families about medical errors that impacted their care	3.5	0.9	4	1	98	50.5
	3	I intend to encourage my colleagues to report any patient safety issues they encounter	3.8	0.8	4	1	128	66.0
	4	I intend to report any patient safety issues I encounter	3.6	0.9	4	1	109	56.2
	5	If I saw a medical error committed by one of my team members, I would keep it to myself^a^	2.7	0.9	4	1	80	41.2
Overall patient safety grade		Overall, based on your clinical rotation, do you think your hospital is safe?^b^	7.4	1.9	8	3	120	61.9

^a^Reverse-coding: 5 (strongly disagree) to 1 (strongly agree), positive responses mean “disagree” and “strongly disagree”

^b^Full marks: 1 (strongly disagree) to 10 (strongly agree)

For the teamwork culture domain, 78% of medical students reported that the quality of care received by patients was impacted by teamwork during clinical rotations. Of the respondents, 40% agreed that it was easy to ask questions when there was something that they did not understand.

Regarding error disclosure domain, positive scores ranged from 10% to 74%. Except for one question asking whether the disclosure of medical errors was an important component of patient safety, the percentages of positive scores for all the other questions were below 20%. In particular, only 10% of respondents agreed that they were encouraged by colleagues to disclose errors to patient and families; this was the lowest score. However, many more (50%) reported that they had the intention to encourage colleagues to tell patients and their families about medical errors.

Independent of survey domains, when students were asked, “Overall, do you think your hospital is safe based on your clinical rotation?” a majority (60%) reported that the hospital was safe. The mean and median of overall patient safety were 7.4 and 8, respectively.

**Fig. 1:**
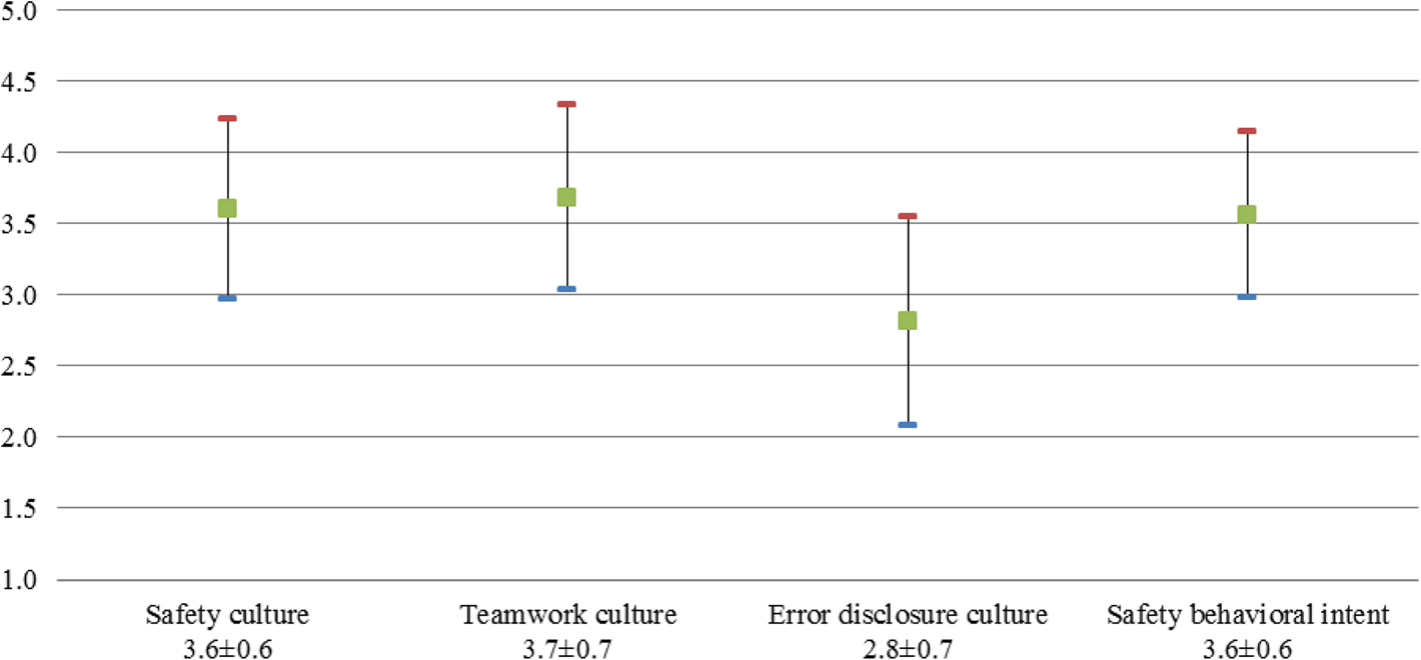
Distribution of overall scores for the four domains

Figure 1 shows the mean of each safety domain. Among safety domains, “teamwork culture” was rated highest. “Error disclosure culture” received the lowest ratings among the assessed safety domains.

### Associations among the three cultural domains, behavioural intentions, and perceptions of overall patient safety

We calculated Spearman correlations among the three cultural domains, the five factors related to behavioural intentions regarding safety, and perceptions of overall patient safety during clinical rotations (Table 3). The three domains, safety, teamwork, and error disclosure, showed statistically significant and positive correlations with all factors related to behavioural intentions regarding safety and overall patient safety. Responses to questions addressing the safety culture were positively correlated with the expectation of participation in quality improvement initiatives (*rho* = 0.41). Ratings for the error disclosure culture were correlated with the intention to encourage colleagues to tell patients and families about medical errors that impacted patient care (*rho* = 0.42). The correlation between overall safety grade and error disclosure culture was weakest (*rho* = 0.17).

**Table 3:** Associations among cultures, behavioural intentions, and overall patient safety

	Spearman’s rank correlation coefficient (95% confidence interval)		
Item	Safety culture	Teamwork culture	Error disclosure culture
Safety behavioural intent			
1. I expect to participate in quality improvement initiatives	0.41 (029, 0.52)	0.29 (0.15,0.41)	0.25 (0.12,0.38)
2. I intend to encourage my colleagues to tell patients and their families about medical errors that impacted their care	0.28 (0.14,0.40)	0.16 (0.02,0.29)	0.42 (0.30,0.53)
3. I intend to encourage my colleagues to report any patient safety issues they encounter	0.28 (0.15,0.41)	0.22 (0.09,0.35)	0.25 (0.11,0.38)
4. I intend to report any patient safety issues I encounter	0.34 (0.21,0.46)	0.14 (0.00,0.28)	0.30 (0.17,0.42)
5. If I saw a medical error committed by one of my team members, I would keep it to myself	0.22 (0.08,0.35)	0.14 (0.00,0.27)	0.17 (0.03,0.30)
Overall patient safety grade			
Overall, based on your clinical rotation, do you think your hospital is safe?	0.44 (0.31,0.54)	0.42 (0.30,053)	0.17 (0.03,0.31)

## Discussion

We need information about medical students’ perceptions and intentions regarding patient safety to develop educational programme to be realistic, practical, and relevant to undergraduate medical students’ experience during clinical clerkship. In this study, we measured medical students’ perceptions of cultural factors, as experienced during clinical rotations, after demonstrating the validity and applicability of MSSAPS. This study also demonstrated that medical students’ safety perceptions were associated with their safety behavioural intent.

Except for one question asking whether medical error disclosure was an important component of patient safety, less than 20% of students offered positive responses to questions about the culture surrounding error disclosure culture, and this domain received the lowest ratings among the four safety domains assessed. This trend was consistent with a previous study [7]. Although a high percentage (73.7%) of students reported medical error disclosure *was* an important component of patient safety, a low percentage (18.0%) of students reported they did receive education about error disclosure. Only 10% of students reported they were encouraged by their colleagues to disclose errors and. Such experiences could lead them to internalise the message that disclosure is not a high priority in their organisation, even when they know the importance of error disclosure. A low percentage of students reported clinical culture made it easy to learn from the errors of others (34.5%). Only 41.2% reported they had intention to disclosure when they saw a medical error committed by another team member (41.2%). This indicates that many students had difficulty speaking up about medical errors.

Despite the focus on error prevention, much less attention has been paid to the process of medical error disclosure. Of the limited research on this topic in Korea, most studies have focussed on nurses. According to a previous study, a significant proportion of doctors expressed negative perceptions of their working units’ patient safety culture. Most doctors did not know how and which medical errors to report [16, 23]. Disclosure is particularly difficult because these delicate conversations require advanced communication skills. Error disclosure guidelines and educational efforts aimed at improving sophisticated communication skills are needed for both physicians and students. Formal disclosure curricula, coupled with supervised practice, are necessary to prepare trainees to independently disclose errors to patients by the end of their training. In the absence of formal curricula, trainees may otherwise learn disclosure skills through the hidden curriculum and direct observation of senior clinicians [23]. Transparent clerkship evaluation policies and non-punitive institutional error reporting systems could increase trainees’ positive perceptions in this regard [24]. A medical error reporting system was introduced in Korea in late 2016. Thus, it is important to evaluate changes in the experiences and perceptions not only of medical professionals but also of medical students with regard to error disclosure since this medical error reporting system was introduced.

Over three-quarters of respondents agreed that they followed standard operating procedures, guidelines, and protocols for the floor and operating room. This could be because the clinical rotations at all three medical schools occurred in hospitals with accreditation by The Korea Institute for Healthcare Accreditation (KOIHA). KOIHA operates accreditation programs for healthcare organisations, which include safety, continuous quality improvement, healthcare delivery systems, and evaluation and clinical indicators. The patient safety domain addresses standard operating procedures, guidelines, and protocols for the floor and operating room, such as guidelines on hand hygiene, preoperative time-outs, and post-operative debriefings.

Students’ perceptions of the safety, teamwork, and error disclosure cultures were correlated with their perceptions of overall patient safety. All three cultural domains were correlated positively with behavioural intentions regarding safety. The error disclosure culture was correlated with the intention to encourage colleagues to tell patients and families about medical errors that impacted their care (*rho* = 0.42). Interestingly, the error disclosure culture was more strongly correlated with the intention to encourage colleagues to speak up about a medical error than with the intention to speak up about a team member’s error when they saw it.

This study has some limitations. First, the date came from 3rd-year undergraduate students enrolled at three colleges of medicine, which may limit the generalizability. Second, the findings may differ across medical schools with the different level of education quality. However, we could not consider medical education quality because of lack of information on how patient safety is incorporated into curricula. Lastly, the responses may have been subject to a social desirability bias. However, this study provides an overview of perceptions and intentions regarding the patient safety culture in Korea. This study also identified critical factors, such as error disclosure, that should be featured in education to improve the patient safety culture. A validated survey measuring students’ experiences with the hidden curriculum would be helpful for understanding how students’ attitudes and beliefs change with increasing clinical experience or curricular interventions.

Medical schools and teaching hospitals should raise awareness about the hidden curriculum and its impact on safety. Teachers and students should be trained together in sessions that emphasise effective communication strategies and allow clinicians to reflect on their everyday practices [5]. Additionally, organisations should facilitate feedback from students by asking for their thoughts and reflections in forums that are guaranteed to be safe and non-punitive.

Safety education remains largely absent from pre-service education in many settings. A survey of 125 medical schools in one high-income country found that only 10% had safety content in elective or required courses and only half of recently published medical textbook editions contained safety information [25]. Patient safety training cannot consist of a static, one-time lecture. Medical students learn patient safety behaviours by observing and imitating peers, residents, and faculty [26]. The value of patient safety may be idealized in lectures, but they are demonstrated and reinforced in the real-life setting of the hospital. For example, although students are taught to wash their hands before tending to each patient, they will only truly learn this if it is reinforced by resident and faculty physician behaviour in the clinical setting [27].

Patient safety has emerged as a global concern in the provision of quality healthcare [9]. Since the Patient Safety Act was implemented in 2016 in Korea, there has been an increasing need to incorporate patient safety principles in medical education, including at the undergraduate level [8]. Some medical schools in other countries have implemented patient safety curricula [28–30]. In Korea, however, a patient safety medical curriculum has not been actively discussed by medical educators [31]. Medical students’ experiences during clinical rotations have an important influence on their attitudes towards patient safety and their future behaviours [7]. More attention to and recognition of patient safety by all healthcare personnel and medical educators is needed. Thus, discussions of medical errors, patient safety, and how best to incorporate an analysis of these issues into the existing curriculum are needed.

## Conclusions

Our study provides evidence that many students had difficulty speaking up about medical errors during clinical rotations, thereby suggesting that error disclosure guidelines and educational efforts aimed at developing sophisticated communication skills are needed. This study may serve as a reference for medical schools or other institutions planning patient safety education in their curricula. Safety culture assessment can highlight opportunities for improvement and contribute to a positive safety culture.

## Abbreviations

CFI: Comparative fit index
CI: Confidence interval
KOIHA: The Korea Institute for Healthcare Accreditation
MSSAPS: Medical Student Safety Attitudes and Professionalism Survey
NFI: Normed Fit Index
NNFI: Nonnormed Fit Index
RMSEA: The Root Mean Square Error of Approximation
